# Metal-Ligand Coordination Induced Ionochromism for π-Conjugated Materials

**DOI:** 10.3389/fchem.2020.589106

**Published:** 2020-10-02

**Authors:** Jinhui Liu, Long Han, Jieting Geng, Jing Hua, Zhaobo Wang

**Affiliations:** Key Laboratory of Rubber-Plastics Ministry of Education/Shandong Provincial Key Laboratory of Rubber-plastics, College of Polymer Science and Engineering, Qingdao University of Science and Technology, Qingdao, China

**Keywords:** sensors, metal ions, π-conjugated polymers, chemosensors, ionochromism

## Abstract

Recent studies indicated that the toxicity of heavy metal ions caused a series of environmental, food, and human health problems. Chemical ionochromic sensors are crucial for detecting these toxicity ions. Incorporating organic ligands into π-conjugated polymers made them receptors for metal ions, resulting in an ionochromism phenomenon, which is promising to develop chemosensors for metal ions. This review highlights the recent advances in π-conjugated polymers with ionochromism to metal ions, which may guide rational structural design and evaluation of chemosensors.

## Introduction

**Figure 1:**
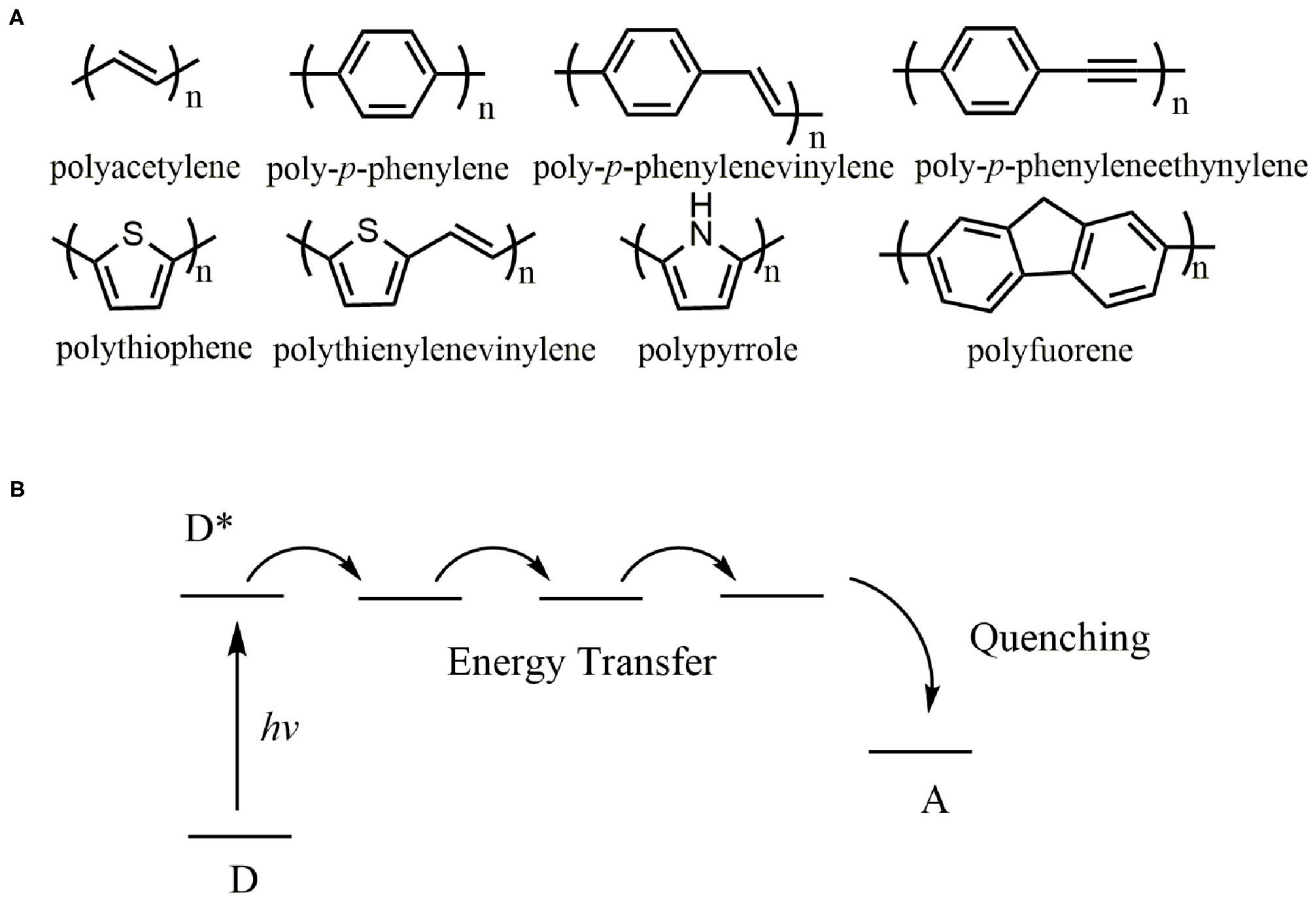
**(A)** Chemical structures of π-conjugated polymers, **(B)** Illustration of energy-transfer quenching through a conjugated polymer. ^*^Excited state.

π-Conjugated polymers refer to polymers in which double bonds (or triple bonds) and single bonds are alternately arranged, including π-π conjugated system, p-π conjugated system, and σ-π-conjugated system (Moon et al., 2007; Tan et al., 2009). It is a research hotspot in the multidisciplinary research field (Bhupathiraju et al., 2018; Biswas et al., 2020; Lee et al., 2020; Zhao et al., 2020) since Shirakawa and coworkers discovered that polyacetylene (PA) doped with I_2_ or AsF_5_ exhibited high conductivity more than 10^3^ S/cm in 1997 (Chiang et al., 1977; Shirakawa et al., 1977). Some parent structures of π-conjugated polymers in the backbone are presented in Figure 1A (Hoeben et al., 2005). By far π-conjugated polymers are the most promising functional polymers in the field of cheap and portable electronic devices such as photovoltaic cells (Brabec et al., 2003; Chen et al., 2019), light-emitting diodes (Tang et al., 2013; Saito et al., 2018), prototype field-effect transistors (Dimitrakopoulos and Malenfant, 2002; Melkonyan et al., 2016; Zhang et al., 2017, 2018; Zhang H. et al., 2020) and coatings (Zeng et al., 2019; Zhang H. C. et al., 2020).

In recent years, researches on the π-conjugated system are booming in the fields of organic optoelectronics and sensors based on chromogenic effect. Chromogenic systems are required to be responsive to external inputs, such as metal ions (ionochromism), (Cheng and Tieke, 2014) electrons (electrochromism), (Thakur et al., 2012), or light (photochromism) (Bisoyi and Li, 2016) that could be used for image production. Among these chromogenic systems, ionochromism is realized through the coordination between metal and ligand, resulting in sensitivity to metal ions, which could be used as metal sensors. Trace metal detection plays a vital role in the environment, the human body, and equipment safety. According to the Environmental Protection Agency (EPA), thirteen heavy metal ions are listed as “priority pollutants” because the toxicity of heavy metal ions caused a series of environmental problems. It is of significance to detect the content of alkali metals ions for human condition monitoring.

This review aims to bring together the areas of metal-ligand coordination and π-conjugated systems. Materials based on metal-ligand coordination that show a polymerlike structure in solid-state refers to “metal-organic frameworks” (MOFs) (Kaneti et al., 2017; Ding et al., 2019), and in solution refers to “metallo-supramolecular polymers” (Winter and Schubert, 2016). MOFs and metallo-supramolecular polymers will not be considered in this review because they have been reviewed a lot in recent years. Therefore, this review focuses on the ionochromic properties of π-conjugated polymers with organic ligand as receptors for metal ions.

## Ionochromism

Ionochromic effect based on π-conjugated polymers generates from induced conductivity fluctuations either by destroying the conjugation of polymers (conformational effect) or by lowering charge carrier mobility (electrostatic effect). The conductivity of π-conjugated polymers is highly sensitive to the nature and regiospecificity of the side chains, resulting in sensory signal amplification through energy-transfer along polymer chains. Figure 1B illustrated the energy-transfer process, in which upon the excitations, the energy may migrate along the polymer backbone due to the conjugation. As a result, the π-conjugated polymers act as a molecular wire, and the conjugated system generates a response more significant than that afforded by a small interaction in an analogous small mono-receptor system (Zhou and Swager, 1995).

Organic ligand containing conjugated polymers are receptors for metal ions. The coordinating interaction between metal ions and ligand causes electrostatic or conformational changes, resulting in an ionochromic effect. Ionochromic performance in π-conjugated systems is expected to find use in portable optical devices for the detection of metal ions and some organic cations. The electron effect and steric hindrance effect of the ligand are selective for the type of metal. The ionochromic phenomenon in the π-conjugated polymer will be introduced according to the type of ligands.

### Crown Ether

Crown ethers were discovered by the Nobel Prize winner Charles Pedersen (Pedersen, 1967) more than 50 years ago. Recent progress in the design and applications of chemosensors based on crown ethers for small molecules has been reviewed (Li et al., 2017). In contrast to small molecules, π-conjugated polymers have enormous advantages for sensing applications in terms of energy migration and facile exciton transport, which improve the electronic communication between receptors. Additionally, polymers could be processed into films exhibiting semi-permeability to ions. Herein, we focus attention on the design of crown ethers containing π-conjugated polymers and their applications in chemosensors.

**Figure 2:**
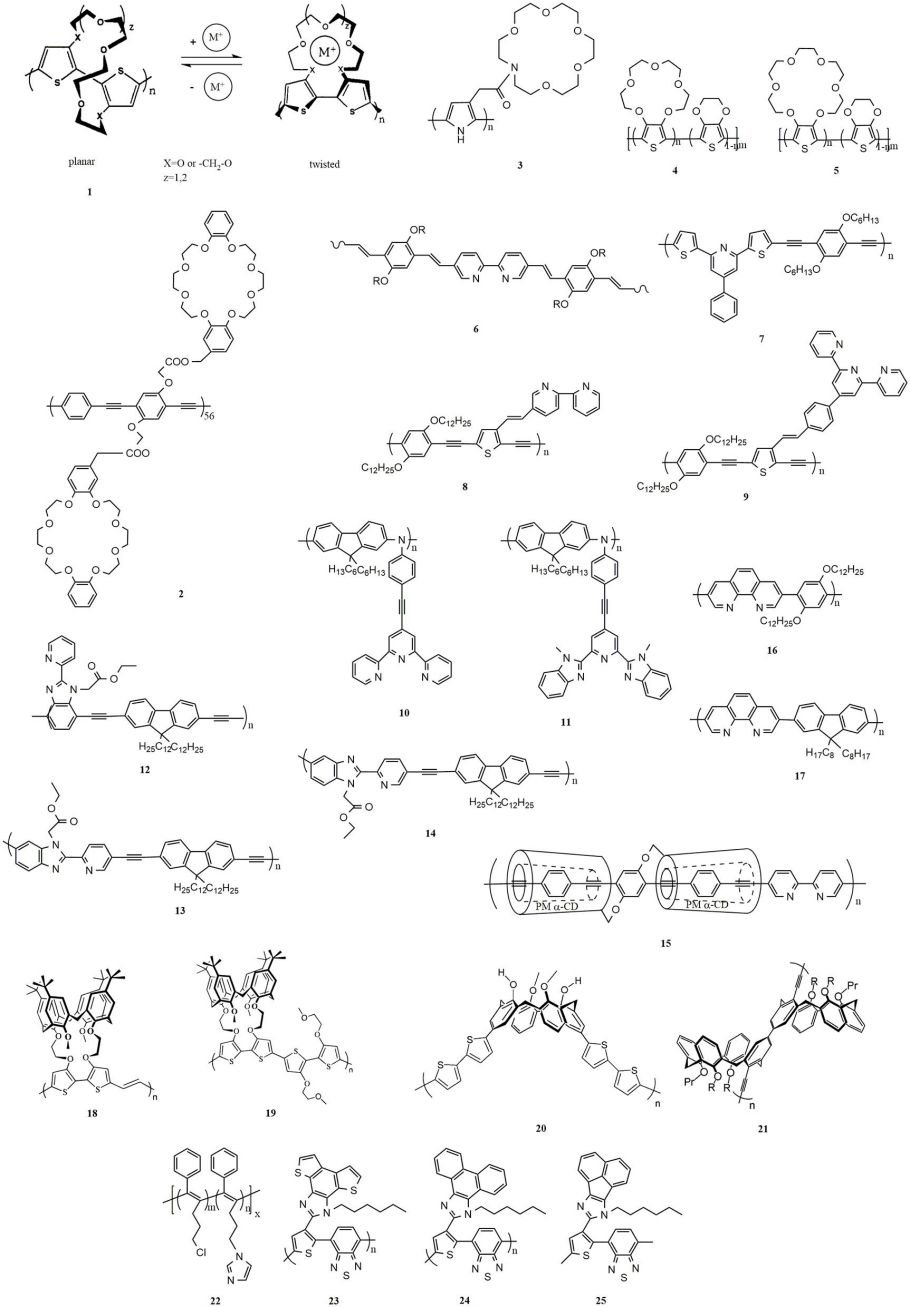
Chemical structures of π-conjugated polymers with ligands.

Ionochromism was initially reported in the 1990s. Upon coordination with alkali-metal ions (K^+^, Na^+^, and Li^+^), polythiophenes with crown ether side chains (**1**, Figure 2) (Bäuerle and Scheib, 1993; Marsella and Swager, 1993) underwent interesting sensory effect because of dramatic conformational changes of polymer chains. Casanovas et al. (Casanovas et al., 2009) studied the affinity of crown ether functionalized polythiophenes for Na^+^, K^+^, and Li^+^ by quantum mechanical calculations. The results showed that although the association of Li^+^ to the polythiophenes derivatives is entropically unfavored, the binding energies increased in the order of K^+^ < Na^+^ < Li^+^. The authors explain that the alkali ions with small dimensions underwent large fluctuations when the dimensions of the cavities changed, leading to an increase in thermal energy.

In addition to polythiophene, chemosensors based on other conjugated systems containing benzocrown or azacrown ethers were also developed. A multiple signal responsive chemosensor was realized by a poly(phenylene-ethynylene) polymer with pendent dibenzocrown groups (**2**), which was responsive to multi-excitation (K^+^, Cl^−^, pH, or temperature change) (Ji et al., 2013). Additionally, exposure of a **2-**based film to ammonia increased fluorescence, making it a good candidate for gas sensing. Morgado et al. studied the ionochromic properties of poly(*p*-phenylenevinylene) (PPV) with benzocrown ether (Morgado et al., 2000). Single-layer devices with Al cathodes showed higher electroluminescence efficiencies than those with Ca cathodes due to the existence of aggregates, induced by the crown ether side groups.

Due to the easy chemical access to modification, the functionalization of polypyrrole is widely studied by incorporating various active groups on the nitrogen atom. However, the modification of polypyrrole generated a loss of conjugation, resulting in extremely low conductivity for poly(N-substituted pyrroles), of the order of 10^−4^ S/cm or less (Eaves et al., 1985; Bettelheim et al., 1987). Youssoufi et al. found that the equivalent 3-substituted pyrroles (**3**) gave rise to highly conducting polymer films, and they developed azacrown ether substituted polypyrroles with selective cation binding on voltammetric cycling in organic media (Youssoufi et al., 1993). In contrast to benzocrown ethers, a drawback of the azacrown ethers is that it exhibits low thermodynamic stability upon alkali and alkaline-earth metal ions (Ushakov et al., 2008). The main reason is that the planar structure of the junction section of the azacrown ether and benzene moieties. The increasing electron-withdrawing ability of the moiety conjugated to the crown ether is helpful for improving the thermodynamic stability. However, this method does not always lead to the expected enhancement of optical signal induced by metal ions but may conversely attenuate the signal (Izatt et al., 1996; Ushakov et al., 1997). Gromov et al. (Gromov et al., 2013) synthesized 4-pyridine-, 2-benzothiazole-, and 2-, and 4-quinoline-based styryl dyes with an n-methylbenzoazacrown ether as a ligand. Electron spectroscopy studies showed that these compounds had a high sensibility for alkali metal and alkaline earth metal cations. In terms of electrochromism and cation binding capacity, they proved to be far superior to those based on phenylazacrown ether. After complexing with Ba^2+^, the fluorescence enhancement factor reached 61. The discovery of high levels of macrocyclic pre-organization is one of the factors that determine the high cation binding capacity of sensor molecules based on N-methylbenzoaza-crown ether.

Alkali-metal ions, especially K^+^ and Na^+^, are the messengers of living cells, controlling a series of physiological processes through the action of ion channels. Crown ether containing π-conjugated polymers are highly sensitive to alkali-metal ions and could be designed to medical detectors. Nevertheless, most researches focused on the ionochromism of sensors operating in organic solvents rather than in aqueous solutions (Xiang et al., 2014), which is impractical for applications. Additionally, most reported ion-selective films require long incubation times to generate a detectable response, precluding their practical use (Giovannitti et al., 2016). Single-component π-conjugated polymers (**4** and **5**) were synthesized that respond selectively and rapidly to varying concentrations of Na^+^ and K^+^ in aqueous media, respectively (Wustoni et al., 2019). Using a miniaturized organic electrochemical transistor chip, variations in the concentration of these two metal ions in a blood serum sample could be measured in real-time. The devices based on these crown ether containing polymers are valuable for analyzing cellular machinery and detecting human body conditions that result in electrolyte imbalance.

### Pyridine

The studies on crown ether substituted π-conjugated polymers have clearly demonstrated the ionochromism in alkali chemosensors. It is well-known that oligopyridine, such as bipyridine (bpy), terpyridine (tpy), and its derivatives exhibit super abilities to coordinate a large number of metal ions. If selecting oligopyridine as ligands for π-conjugated polymers, the range of metal ion sensors could be extended from alkali metal ion selective systems to transition metal ion selective systems. Additionally, pyridine and its derivatives not only have the electron-accepting ability to coordinate with metal ions, but also are reactive for metal complex-forming reactions, such as N-oxidation, N-protonation, and quaternization with RX, which can adjust their optical and electrical properties (Yamamoto et al., 1994).

In 1997, Wang et al. reported a transition metal-induced ionochromic polymer with bpy in the backbone (**6**) (Wang and Wasielewski, 1997). According to theoretical calculations, there is a 20° dihedral angle between two adjacent pyridyl rings in bpy when it is in its transoid-like conformation (Cumper et al., 1962). As a result, bpy-based polymers are pseudoconjugated. With the addition of metal ions, the chelating effect of bpy ligand with the metal ions forces the pseudoconjugated conformation into a planar one, and thus makes the polymers fully conjugated, leading to the redshift in absorption spectra. Besides, incorporating bpy as a ligand directly into the backbone results in a more sensitive response with the addition of metal ions. Different linkers between bipyridine and conjugated polymer in the backbones cause differences in flexibility and rigidity of the resulting polymers. Bin Liu et al. studied the effect of linkages, including C-C single, vinylene, and ethynylene bonds, on the electronic properties and response sensitivities to metal ions (Liu et al., 2001). During the chelation with metal ions, the C-C single bond linkage provided better flexibility to the coplanarity of the pyridine unit. Therefore, C-C single bond possessed the highest sensitivity, and it was followed by vinylene bond, while ethynylene bond exhibited the lowest sensitivity. A conjugated polymer containing 2,6-substituted pyridine derivative (**7**) was synthesized for Pb^2+^ sensing (Liu et al., 2011). With the addition of Pb^2+^, the color changed from yellow-green to brown, and this can be easily observed by the naked eye. The detection limit of the polymer is less than 1 ppm, while the threshold of Pb^2+^ in drugs is 5–10 ppm. Therefore, **7** could be adopted to design an excellent sensor for Pb^2+^ detection.

Increasing the association constant of a molecular recognition event could improve the sensitivity of a sensor. Terpyridine (tpy) ligand possesses an excellent ability to coordinate various of metal ions with higher sensitivity than bipyridine. Zhang et al. prepared poly[p-(phenyleneethynylene)-alt-(thienyleneethynylene)] (PPETE) with bpy (**8**) and tpy (**9**) as receptors, respectively (Zhang et al., 2002). With the addition of Ni^2+^, **9** was quenched to 10.9% of its original emission intensity, while **8** was only quenched to 38.2%, illustrating that tpy was more sensitive to Ni^2+^ ion. Rabindranath et al. reported tpy substituted polyiminofluorenes (**10**) (Rabindranath et al., 2009). Fe^2+^, Co^2+^, Ni^2+^, Cu^2+^, Pd^2+^, Fe^3+^, Gd^3+^, and Zr^4+^ led to complete quenching of the green emission for **10**, while Zn^2+^, Cd^2+^, and Eu^3+^ caused a weak red emission, which was redshifted by 10-30 nm compared with pure **10**. Additionally, a biscomplex formed upon the addition of Zn^2+^, leading to red luminescent precipitation. This effect can be used for the detection of Zn^2+^.

Although tpy exhibits high coordinating ability with a large number of metal ions, the preparation of tpy ligands is expensive and very time-consuming. In contrast to bpy, 2,6-bis(10-methylbenzimidazolyl)pyridine (bip) ligands can be more easily synthesized on a large scale (Beck et al., 2005). Kalie Cheng et al. reported a polyimide fluorene (**11**) with bip ligand (Cheng and Tieke, 2014) and studied the optical properties with Zn^2+^ and Cu^2+^. Due to charge transfer from ion-specific metal to ligand, the **11**/Zn^2+^ film is orange, while the **11**/Cu^2+^ film is purple. Due to the oxidation of the polymer backbone, the **11**/Zn^2+^ and **11**/Cu^2+^ films will turn blue if anodically oxidized to 750 mV vs. FOC., and the color change is reversible. The conjugated polymer with bip ligand exhibited high contrast and short switching times in color change upon 12 dipping cycles. However, the long-term stability of bip with metal ions is lower than that of tpy based systems.

Some biomacromolecules such as DNA, RNA, and proteins are easy to be inhibited by Pd^2+^ *in vivo* and *in vitro* (Kielhorn et al., 2002). Additionally, Pd^2+^ is able to elicit a series of cytotoxic effects, resulting in severe primary skin and eye irritations. It is essential to investigate a sensor for highly selective and sensitive detection of Pd^2+^. Xiang et al. reported three conjugated polymers (**12–14**) *via* Sonogashira reaction (Xiang et al., 2014). The conjugated backbones of **13** and **14** are twisted, which were proved to be selective for Ni^+^. In contrast to **13** and **14**, **12** exhibited high selectivity for Ag^+^ because of its linear conjugated backbone. Theoretically, the same functional group should have the same metal ion recognition capability. According to the ionochromic effect of **12**–**14**, changes of linkage site for recognition groups resulted in different metal ion selectivity. Cyclic voltammetry measurement for **12–14** was carried out to analyze the cation selectivity by LUMO and HOMO energy levels. The LUMO levels of **12** are slightly lower than that of **13** and **14**, indicating that their electron affinity is in the order of **12** > **14** ≈ **13**. Additionally, the HOMO levels of **13** and **14** are slightly raised relative to **12**, illustrating that the energy barrier of hole injection from the anode is in the order of **13** = **14**<**12**. As a result, both electron and hole affinities of **12** are improved, resulting in enhanced carrier injection and transport. Moreover, smaller coordination cavity in **13** and **14** fits well with the size of Ni^+^ because of their twisted conjugated backbones and smaller radius of Ni^+^. This work provided guidelines to tune the structure of conjugated polymers for the design and preparation of the selective metal ion sensors.

Despite the successful development of chemosensors in conjugated polymers, most of the examples are in the solution state, and seldom chemosensors in neat π-conjugated polymer films have been reported. This is because of strong interpolymer π-π interactions resulting in the self-quenching of luminescence in such a condensed solid-state phase (Sahoo et al., 2014). It is a non-negligible challenge to control such random and strong interactions in the solid-state. Hosomi et al. reported π-conjugated polymer with bipyridine moieties as ligand and permethylated α-cyclodextrin (PM α-CD) as the main chain (**15**). The PM α-CD suppresses the interactions between π-conjugated and enabled the polymers to show efficient emission even in the solid-state (Hosomi et al., 2016). Additionally, the metal-ion recognition ability of **15** is maintained in the solid-state, leading to reversible changes in the luminescent color in response to cations. The prepared π-polymer is expected to be applicable for recyclable luminescent sensors to detect different metal ions.

### 1,10-Phenanthroline

1,10-Phenanthroline (phen) is an electron-poor, rigid planar, hydrophobic, and heteroaromatic ligand that has played an important role in the development of coordination chemistry (Cockrell et al., 2008; Bencini and Lippolis, 2010; Iqbal et al., 2016). Phen is a bidentate ligand for transition metal ions whose nitrogen atoms are beautifully placed to act cooperatively in cation binding. In contrast to the parent bpy and tpy systems, phen is characterized by two inwardly pointing nitrogen donor atoms, which is held juxtaposed. As a result, phen is pre-organized for strong and entropically favored metal binding. A luminescent phen-containing π-conjugated copolymer (**16**) responsive with Zn^2+^, Ir^3+^, and Eu^3+^ was reported (Yasuda et al., 2003). The λ_max_ of **16** is shifted from 385 to 404 nm on the addition of NiCl_2_. Photoluminescence intensity of 1 steeply decreases in the presence of Ni^2+^ because of the chelating effect of the phen unit to Ni^2+^. Other metal ions also caused similar shifts of λ_max_. Mainly, Li^+^, Mg^2+^, Al^3+^, Zn^2+^, Ag^+^, and La^3+^ caused a redshift of λ_max_ to a smaller degree of about 10 ± 35 nm; Fe^3+^, Co^2+^, Ni^2+^, Cu^2+^, and Pd^2+^ gave rise to a larger redshift by about 20 ± 50 nm and complete quenching of photoluminescence. This quenching phenomenon is related to an energy transfer from the π-conjugated polymer to the metal complexes. Yasuda group further synthesized a copolymer composed of alternating phen/9,9-dioctylfluorene (**17**) (Yasuda and Yamamoto, 2003). The color of emitted light from the polymer complex could be tuned from blue to red by transition metal ions (Co^2+^, Ni^2+^, Cu^2+^, and Pd^2+^) upon absorption spectra. Additionally, Satapathy et al. reported conjugated polymers containing phenanthroline that show remarkable sensing capabilities toward Fe^2+^ (Satapathy et al., 2012).

### Calixarene

Calixarenes have unique hole structures, which can be functionalized to recognize metal ions. Moreover, the hydrophobic cavity of the calixarene scaffold can accommodate various gases and organic molecules (Rudkevich, 2007). With the addition of metal ions or small molecules, calixarenes undergo dramatic geometric changes, including phenol ring flips between cone, partial cone, and 1,3-alternate conformational isomers (Gutsche, 1998). The small molecular of calixarene-based sensors for recognition of transition metal cations have been recently reviewed (Kumar et al., 2019), and here only list some calixarene-based conjugated polymer sensors. Calixarene-functionalized polymers (**18** and **19**) were first reported in the 1990s (Marsella et al., 1995). Binding constant measurements of the calixarene-bithiophene generated a Ka (7.6 × 10^7^) for Na^+^, which is approximately100 times stronger than K^+^ and 40 times stronger than Li^+^. A stronger binding constant means higher sensitivity. Ion recognition behavior of **18** and **19** toward Li^+^, Na^+^, and K^+^ was analyzed by UV-vis absorption and fluorescence emission spectroscopy. The resulting polymers exhibit good selectivity toward Na^+^, with a 24 nm blue shift for 66 and 32 nm red shift for 67.

Other calixarene-based receptors in π-conjugated polymers were reported (Wosnick and Swager, 2004; Costa et al., 2008), in which the calixarene groups were mainly as pendant groups. The direct attachment of the calixarene unit (at the upper rim) to a conjugated polymer (**20**) has also been reported (Yu et al., 2003). The conical configuration of calixarene makes the polymer chain segment a zigzag orientation. The segmental structure in **20** imposes great localization of the carriers, and the rapid self-exchange between discrete units causes the conductivity of such a segmented system. Protonation promoted the electron exchange resulted in high conductivity for **20**. Hence, electroactive calixarene polymer that requires protonation to be highly conductive was prepared, which is useful for the design of actuating materials. A fluorescent polymer (**21**) in which calixarene scaffolds are the part of uninterrupted linear polymeric backbone was first reported (Molad et al., 2012). Short conjugated fragments combined with the nonlinear geometry gave rise to rather moderate sensitivity with selected stimuli. The coordination of the calixarenes in the π-conjugated polymers allows for the recognition of small molecules, such as NO.

### Imidazole

Imidazole-based ligands are widely used due to their reversible fluorescence. This reversibility is realized by protonation/deprotonation upon an acid/base or metallation/demetallation with metal ions/suitable counter ligands (Jiang et al., 2010). As an important functional conjugated polymer material, polydiacetylene (PDA) has received more and more attention since the first report in 1969 (Wegner, 1969). PDA has significant color conversion and fluorescence enhancement under various environmental stimuli, including heat (Takeuchi et al., 2017), organic solvent (Yoon et al., 2007), bioanalyte (Zhou et al., 2013), ion (Wang et al., 2016), and so on. In response to different stimuli, PDA can be changed to different colors, such as purple, yellow, orange, or red, of which the transition from blue to red is the most common type. Due to their spontaneous color change and fluorescence emission development under stimulation, many PDA liposomes with specific receptor groups have been designed and widely used to detect metal ions such as Hg^2+^ and Cu^2+^ (Lee et al., 2009; Xu et al., 2011).

An imidazole-functionalized disubstituted polyacetylene (**22**) was prepared (Zeng et al., 2008). **22** was not sensitive to alkali and alkaline earth metal ions, and transition metals Cd^2+^, Mn^2+^, Ag^+^, and Zn^2+^, because of the poor coordination ability of the imidazole receptor with these ions. Nevertheless, Pb^2+^, Al^3+^, and Cr^3+^ could quench the fluorescence of **22** not completely, while Cu^2+^, Co^2+^, Fe^2+^, Fe^3+^, and Ni^2+^ could quench its fluorescence more efficiently. Particularly, Cu^2+^ quenched the fluorescence entirely at a very low concentration (1.48 ppm). Satapathy et al. reported imidazole-based polymers (**23-25**) that present significant ion recognition ability toward Fe^2+^ in semi-aqueous solutions (Satapathy et al., 2012). The fluorescence lifetime of polymer **25** (11.4-fold) decreased larger than that of **23** (4.6-fold) and **24** (6.2-fold) further, illustrating that **25** showed superior sensing capability by virtue of its stronger molecular wire effect. The fluorescence of these three polymers recovered by adding phenanthroline or Na_2_-EDTA. Additionally, the selectivity of **23**-**25** for Fe^2+^ interaction was not interfered by other competing metal ions.

## Conclusions and Outlook

π-Conjugated polymers represent useful chemical platforms for the design of chemosensors for metal ions. In this review, we have summarized the types and characteristics of functional groups that chelating with different metal ions as well as the ionochromic effect of the π-conjugated polymers based on these functional groups. In the past few decades, significant progress has been made in the development of novel chemosensors in environmental protection, food and drug testing, and human health monitoring. Although there has been a lot of research on these materials, preparing chemical sensors with high sensitivity, long-term stability, and selectivity is still a critical challenge. The chemical and physical relationship between ligand and metal coordination also needs to be further studied to improve the theoretical guidance for the preparation of metal sensors.

## Author Contributions

JL and JH: conceptualization and design. LH and ZW: acquisition of data. JG: software. JL and LH: analysis of data. JL: drafting of article. JL, LH, JG, JH, and ZW: final approval of manuscript. All authors: contributed to the article and approved the submitted version.

## Conflict of Interest

The authors declare that the research was conducted in the absence of any commercial or financial relationships that could be construed as a potential conflict of interest.
